# Longitudinal Study of Therapeutic Adherence in a Cystic Fibrosis Unit: Identifying Potential Factors Associated with Medication Possession Ratio

**DOI:** 10.3390/antibiotics11111637

**Published:** 2022-11-16

**Authors:** Rosa Mª Girón, Adrián Peláez, Amparo Ibáñez, Elisa Martínez-Besteiro, Rosa Mar Gómez-Punter, Adrián Martínez-Vergara, Julio Ancochea, Alberto Morell

**Affiliations:** 1 Servicio de Neumología, Instituto de Investigación Sanitaria de La Princesa, Diego de León nº62, 28006 Madrid, Spain; 2 Facultad de Medicina, Universidad Autónoma de Madrid, 28049 Madrid, Spain; 3 Centro de Investigación en Red de Enfermedades Respiratorias (CIBERES), Instituto de Salud Carlos III (ISCIII), 28029 Madrid, Spain; 4 Servicio de Farmacia Hospitalaria, Hospital Universitario de La Princesa, 28006 Madrid, Spain

**Keywords:** cystic fibrosis, therapeutic adherence, CFTR modulators, antibiotics, MPR, machine learning, random forest

## Abstract

Cystic fibrosis (CF) is a genetic and multisystemic disease that requires a high therapeutic demand for its control. The aim of this study was to assess therapeutic adherence (TA) to different treatments to study possible clinical consequences and clinical factors influencing adherence. This is an ambispective observational study of 57 patients aged over 18 years with a diagnosis of CF. The assessment of TA was calculated using the Medication Possession Ratio (MPR) index. These data were related to exacerbations and the rate of decline in FEV_1_ percentage. Compliance was good for all CFTR modulators, azithromycin, aztreonam, and tobramycin in solution for inhalation. The patients with the best compliance were older; they had exacerbations and the greatest deterioration in lung function during this period. The three variables with the highest importance for the compliance of the generated Random Forest (RF) models were age, FEV_1_%, and use of Ivacaftor/Tezacaftor. This is one of the few studies to assess adherence to CFTR modulators and symptomatic treatment longitudinally. CF patient therapy is expensive, and the assessment of variables with the highest importance for a high MPR, helped by new Machine learning tools, can contribute to defining new efficient TA strategies with higher benefits.

## 1. Introduction

Cystic fibrosis (CF) is a genetic and multisystemic disease that requires significant therapeutic demands for its control, both in terms of nutritional recommendations and physical activity, as well as compliance with multiple treatments, with an estimated daily average of up to seven [1]. The planned treatment requires several hours a day and different routes of administration—oral, inhaled, sometimes subcutaneous, or intravenous—altering the routines and activities typical of the age group, which constitutes an important therapeutic burden [1,2].

Until a few years ago, the existing therapies were only aimed at alleviating the symptoms caused by the disease: physical exercise, physiotherapy, bronchodilators, hypertonic or mucolytic substances to improve mucociliary clearance, immunomodulators, and antibiotics to control inflammation and bronchial infection, dietary recommendations, pancreatic enzymes, and vitamin supplements to maintain a good nutritional status [3]. In recent years, CFTR (transmembrane conductance regulator protein) modulation therapy has been developed, including correctors such as Lumacaftor, Tezacaftor, and Elexacaftor and enhancers such as Ivacaftor, which respectively repair and activate chloride channel function, improving the characteristics of secretions from all organs [4,5,6,7,8]. These treatments are currently indicated in patients heterozygous or homozygous for the F508del mutation and are leading to a major change in the lives of these patients with minimization of most symptoms, which may result in a reduction or abandonment of symptomatic treatment [4,5,6,7,8].

In CF, therapeutic adherence (TA) is essential for controlling the disease, preventing exacerbations, and slowing progression [2]. TA is the degree to which a patient’s behavior conforms to the recommendations agreed with the healthcare professional, including medication intake, diet, or lifestyle habits [9]. Non-compliance with the therapeutic regimen has multiple clinical and economic consequences, judging by the increased mortality or morbidity observed in non-compliant patients [10,11,12,13]. Therefore, monitoring TA is relevant in severe chronic diseases such as CF.

Adherence questionnaires are generally unreliable in CF compared to other methods because they overestimate adherence [14,15]. The most used method is the Medication Possession Ratio (MPR). Different studies have shown that the average adherence rate in CF between school age and adulthood is around 50%, with a variation between 48% and 63% [2,10,11,12,13]. It has been observed in different published studies of TA in CF that adherence is worse when the disease is perceived as mild or severe and better when it is moderate [2], that a functional and well-structured family is associated with better adherence [2], while parental anxiety and depression are associated with worse TA [2,16]. In addition, lack of time is an important cause of non-adherence, with better adherence from Monday to Friday [2]. Moreover, TA levels vary depending on the type of treatment. In general, adherence rates are highest for oral antibiotic treatments, lowest for nebulized therapies and pancreatic enzymes, and are lower for vitamin therapy, dietary changes, exercise, and respiratory physiotherapy [10,11,12,13].

Due to the high availability of medical and health information provided by portable networked devices, the use of health data for research purposes is becoming increasingly common [17]. In medicine, an important task is predicting a categorical response variable based on a large number of predictor variables. One of the key objectives of this research is to reliably identify relevant predictors from a large set of candidate variables. A small sample size limits this approach; health data sets usually contain a small number of subjects with the right characteristics to be compared. Traditional statistical techniques used for this purpose, such as logistic regression, are completely limited by this problem [18]. New machine learning tools used for predicting and selecting relevant variables can provide robust results for data limited by sample size, which have achieved great popularity in recent years in many areas [19]. Recently, the use of the RF method has received enormous attention because it can handle many predictor variables with a small number of observations [20]. Predicting the most important variables in TA will allow us to carry out personalized adherence treatments that are more efficient than those already in place.

The study’s hypothesis is that CF is a multisystem disease requiring complex, time-consuming treatments for patients, so a low TA is assumed. Poor TA will have consequences on the clinical course. The aim of this study was to evaluate longitudinally, in a population of adult patients with CF, how adherence to the different treatments has been over the last 2.5 years, to study, in relation to the degree of TA the clinical consequences in terms of lung function and the number of respiratory exacerbations, and to assess the possible predictors of adherence.

## 2. Results

### 2.1. Demographic and Clinical Characteristics

**Figure 1:**
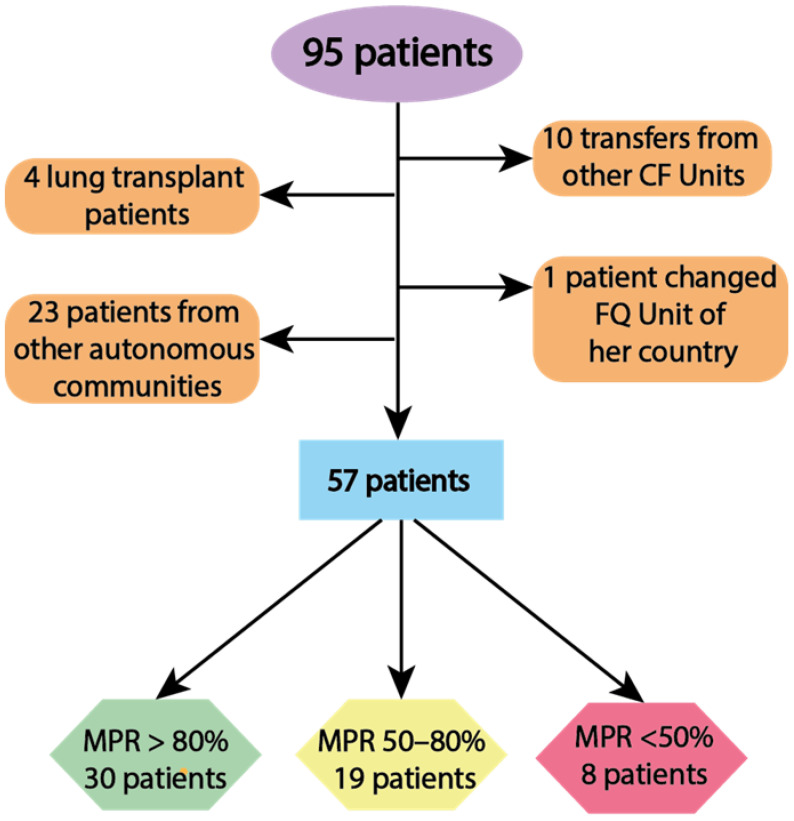
Flowchart of participants, classifying patients according to their Medication Possession Ratio (MPR) percentage. The patients assessed for this study were stratified into three subgroups (MPR > 80%, MPR 50–80%, MPR < 50%) according to their MPR% for all treatments followed throughout the study.

Our study started with a total of 95 patients, of which 38 patients were discarded (10 had been transferred from other CF units recently, four were patients who had previously received a lung transplant, one patient changed CF unit to her country, and 23 were patients from other autonomous communities), resulting in a total of 57 patients to evaluate. Patients evaluated in this study were analyzed according to the mean compliance for all treatments followed throughout the study, classified into patients with high or MPR > 80% (*n* = 30; 49.2%), moderate or MPR50–80% (*n* = 19; 31.2) and low or MPR < 50% (*n* = 8; 13.1%) (Figure 1).

The mean age of all patients evaluated was 33.9 ± 9.4 years, 59.6% female, 35.1% homozygous F508del, 80.7% with pancreatic insufficiency, 42.1% with diabetes, 24.6% with allergic bronchopulmonary aspergillosis and 15.8% with massive hemoptysis. Lung function at baseline, as assessed by the mean percentage of Forced Expiratory Volume in 1 s (FEV_1_%) and the mean percentage of Forced Vital Capacity (FVC%), was 71.0 ± 20.3 and 82.7 ± 15.6, respectively. Chronic bronchial infection by *Pseudomonas aeruginosa* was presented in 22 patients (38.6%), and 16 (28.1%) and 10 (17.5%) for methicillin-sensitive *Staphylococcus aureus* (MSSA) and methicillin-resistant *Staphylococcus aureus* (MRSA), respectively (Table 1).

Patients with low compliance (MPR < 50%) had a statistically significantly lower mean age (*p* = 0.030), mainly for the age range 20–30 years (*p* = 0.017). Gender, Body Mass Index (BMI), and genotype type were similar in the three groups, being the most compliant group (MPR > 80%), the group with the highest proportion for females (63.3%), and F508del homozygotes (46.7%). In addition, the most compliant group had a higher proportion of patients with pancreatic insufficiency (25; 83.3%), allergic bronchopulmonary aspergillosis (9; 30.0%), and massive hemoptysis (6; 20.0%), although this difference was non-statistically significant. Lung function showed a trend of improvement for all three groups during the study, although a non-statistically significant differences were observed between them. The group with the highest compliance had the lowest FEV_1_% and FVC values each year and overall. For chronic bronchial infection, a non-statistically significant difference was observed except for *Haemophilus influenzae* (*p* = 0.004), which was higher for patients with low compliance (4 patients: 50.0%) (Table 1).

The mean number of exacerbations and number of days with exacerbations were assessed for both oral and intravenous cycles of antibiotic treatment. During the study period (2.5 years), for oral cycles, there was a mean total number of exacerbations of 1.5 ± 1.1, and for intravenous cycles, it was 0.2 ± 0.3 (Table 2). The mean number of exacerbations with oral and intravenous cycles decreased from 2.1 ± 1.9 to 0.9 ± 1.0 for the oral cycle and from 0.4 ± 0.8 to 0.0 ± 0.1 for the intravenous cycle. The number of exacerbations for the oral cycle decreased from 43 patients (75.4%) with at least one exacerbation in the first year to 31 (54.4%) in the last year. A similar trend was observed for exacerbations with intravenous cycles, from 14 patients (24.6%) in 2019 to 1 (1.8%) in 2021. The mean number of days decreased over the study years from 47.9 ± 32.5 to 29.5 ± 18.6 for the oral cycle and 26.6 ± 14.0 to 14.0 days for the intravenous cycle.

Regarding the statistical evaluation of the resulting change, only mean values for full years (2019, 2020) were considered. Therefore, months in the year 2021 were excluded to avoid potential biases. The decreasing trend was only statistically significant for the reduction in the number of days assessed per intravenous cycle between 2019 and 2020 (*p* > 0.05).

### 2.2. The Relationship between Compliance and Lung Function

**Figure 2:**
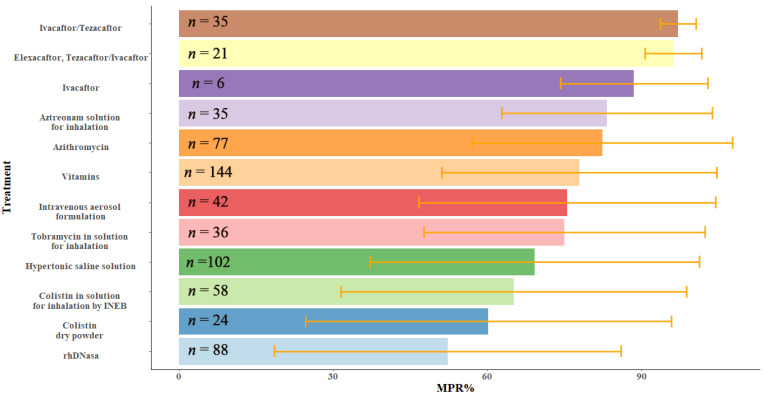
Compliance was measured by % of MPR for each treatment, ranked from highest to lowest mean %MPR. Bars and whiskers represent the mean value and the standard deviation of MPR % for each treatment. The sample size of each treatment appears inside the bar.

Mean compliance was assessed for each type of treatment (Figure 2). All treatments showed compliance above an MPR of 50%. Among all the treatments evaluated, those with the best compliance were those administered orally (Ivacaftor/Tezacaftor, Elexacaftor/Tezacaftor/Ivacaftor, Ivacaftor, Azithromycin), except for vitamins which had a mean MPR value of 77.9% ± 26.7%. In contrast, the treatment with the poorest compliance was one of the secretion fluidizing treatments, rhDNase, with a mean MPR% value of 52.3–33.7%. Within the group of aerosol antibiotics, colistin powder solution and inhalation solution were the second worst compliant treatments, with MPR% of 60.2% ± 35.6% and 65.1% ± 33.6%, respectively.

**Figure 3:**
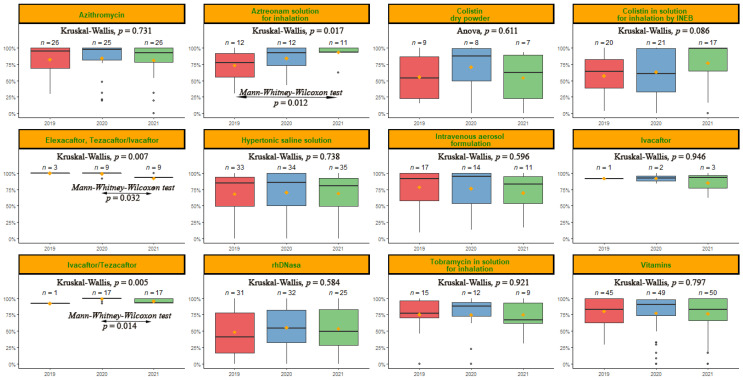
Compliance measured by MPR% for each treatment per year. The test used and the *p*-value obtained after comparing the groups are indicated above each graph. In those groups where a statistically significant difference (*p* < 0.05) was obtained, a post-hoc test was conducted. The type of post-hoc test conducted and the *p*-value obtained from those statistically significant comparisons are indicated between arrows.

The mean compliance for each treatment type was assessed each year (Figure 3). All treatments showed compliance for each of the years assessed, higher than an MPR of 50%, except for rhDNase, which in 2019 had an MPR of 48.2% ± 34.6%. The treatments assessed per year followed a similar trend to when they were assessed in total, with the best compliant treatments being those administered orally, except for vitamins. The worst compliers continued to be rhDNase and colistin.

**Figure 4:**
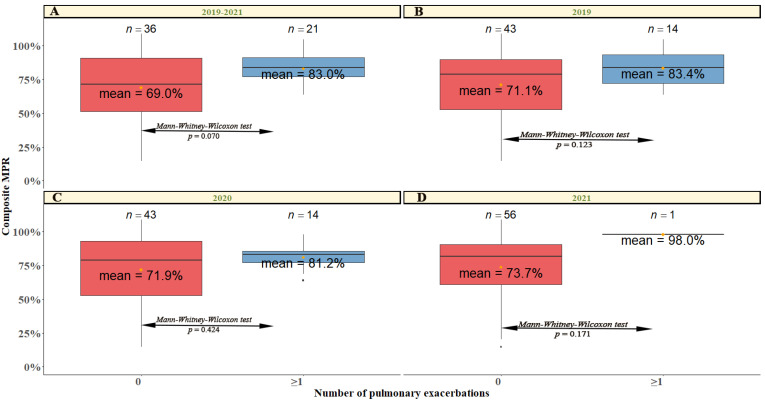
Percentage of MPR for the group of patients who had no exacerbation (0) versus the rest (≥1) in 2019 (panel (**A**)), 2020 (panel (**B**)), 2021 (panel (**C**)), and for all three studies in total (panel (**D**)). The type of test conducted and the *p*-value obtained are indicated between arrows.

When we evaluated the mean MPR% of those patients that required intravenous cycles and presented 0 exacerbations against those who had at least one, we observed that the compliance was superior for those patients with at least one exacerbation (Figure 4A). Although this difference was non-statistically significant (Kruskal-Wallis rank sum tests: Sq *χ*^2^_(1)_ = 3.133, *p* =0.077). This trend continued throughout all years of the study (Figure 4B–D).

**Figure 5:**
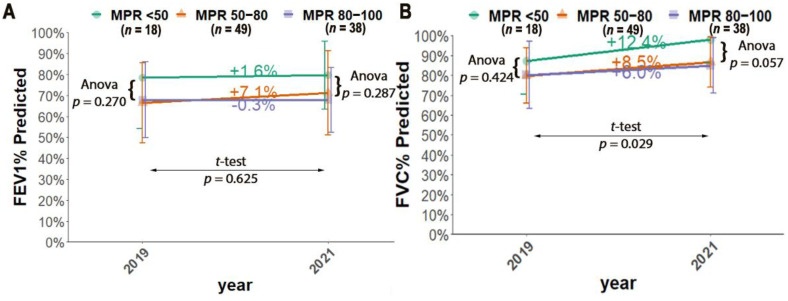
Lung function, FEV_1_ (panel (**A**)) and FVC (panel (**B**)) over time by MPR category for begin and end year of the study (MPR < 50% (2019; n = 10, 2021; *n* = 8), MPR 50–80% (2019; n = 24, 2021; *n* = 25), MPR 80–100% (2019; n = 17, 2021; *n* = 21). The type of test conducted and the *p*-value obtained are indicated between arrows.

When we analyzed which group, based on their MPR% (MPR > 80%, 50–80%, and <50%), had better lung function (Figure 5), we found that the group MPR < 50% had better lung function for both FEV_1_% and FVC%, although this difference was not statistically significant (*p* ≥ 0.057), the lung function of MPR <50% group showed a positive percentage change between the first and last year of the study for FEV_1_% and FVC% (+1.6%, +12.4%, respectively). The MPR 50–80% group had an increase in percentage change (FEV_1_: +7.1%; FVC: +8.5%). However, the group MPR > 80% decreased its percentage change for FEV_1_% (−0.25%) and improved for FVC% (+6.0%). The three groups’ percentage change was not statistically significantly different for FEV_1_ (*p* = 0.625) but for FVC% (*p* = 0.029).

### 2.3. Analysis of Variables with Higher Relevance in the Percentage of MPR

In order to assess which variables have the most predictive power to the response variable and due to the RF method can handle a large number of predictor variables with a small size of observations [20], the prediction of the most important variables in TA was conducted using RF. RF model was performed for a set of 36 variables that could potentially be good predictors of MPR%. To analyze which variables have more relevance in compliance, a model took those patients with an MPR >80% as response variables and another model with a responsible variable MPR < 50%. For the model generation we used the following settings that were previously estimated as the most suitable for our dataset: number of trees (ntree) = 200 and number of variables randomly sampled as candidates at each split (mtry) = 7 [20].

Validation was estimated internally during the algorithm’s execution using the out-of-bag (OOB) method, which measures the prediction error of random forests using Bootstrap aggregation [21]. The assessment of the yielding of the training dataset showed an overall error rate of 30.4% and 12.3%, with an accuracy of 69.6% and 87.7% for the >80% and <50% compliance model, respectively.

**Figure 6:**
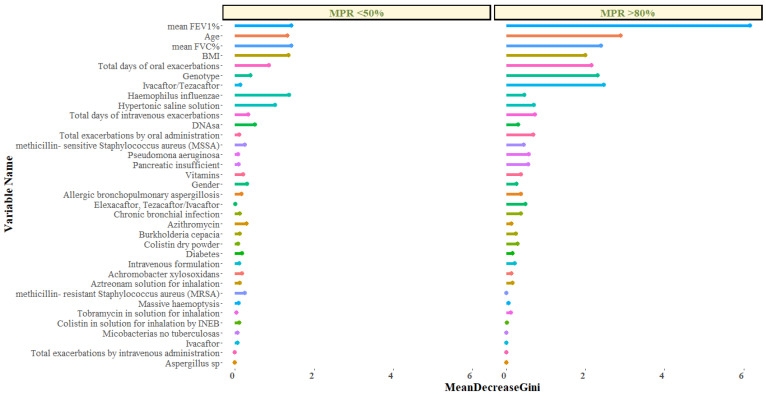
RF plot showing relevant predictors of MPR > 80 (left panel) and MPR < 50 (right panel) using Mean Decrease Gini index (MDG). A higher increase in Mean Decrease Gini corresponds to higher importance in the model.

The RF analysis provides the Mean Decrease Accuracy (MDA) and Mean Decrease Gini (MDG) indices of the predictors evaluated in each of the models (Table 3; Figure 6). We mainly use the MDG index to assess the importance of the variables against the response variable, as the MDG-based rankings provide more robust results than those provided by MDA. Thus, higher MDG values imply that they are predictors with higher significance in the model [20,21].

For the model that uses high compliance (MPR > 80%) as a response variable, the top five relevant predictors were mean FEV_1_% (MDG: 3.3), use of Ivacaftor/Tezacaftor treatment (3.0), age (MDG: 3.0), total days of oral exacerbations (3.0), presence of methicillin-sensitive *Staphylococcus aureus* (MSSA) (2.5). Similarly, for the model using non-compliance (MPR < 50%) as a response variable, the main predictors were mean FEV_1_% (MDG: 1.4), mean FVC% (1.4), BMI (1.4), presence of *Haemophilus influenzae* (1.4), BMI (1.5), age (1.3).

**Figure 7:**
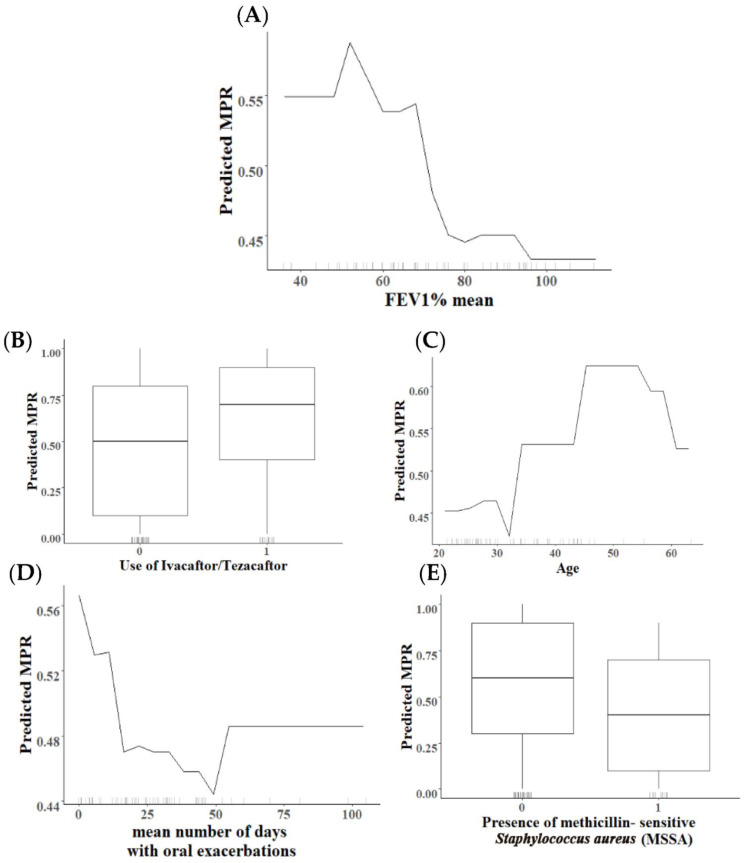
Partial dependence plots for the top 5 most important factors (Panel (**A**): FEV1% mean, panel (**B**): use of Ivacaftor/Tezacaftor, panel (**C**): Age, panel (**D**): mean number of days with oral exacerba-tions, panel (**E**): and presence of methicillin-sensitive *Staphylococcus aureus*) for high compliance.

Besides analyzing which characteristics were most important for explaining the response variables in each generated model (i.e., highest MDG values), we are interested in how the characteristics of these variables influence the predicted outcome. Partial dependence plots (PDP) were constructed for the factor variables with the highest relevance to characterize the relationship between the characteristics of the factors with the highest importance and the %MPR (Figure 7). Each PDP plot reflects the predictions (*y*-axis) for a data point of the characteristics of the individual variables. The marks on the *x*-axis reflect the distribution of the assessed feature, showing how relevant a region is for interpretation (few or no points mean that we should not over-interpret this region).

**Figure 8:**
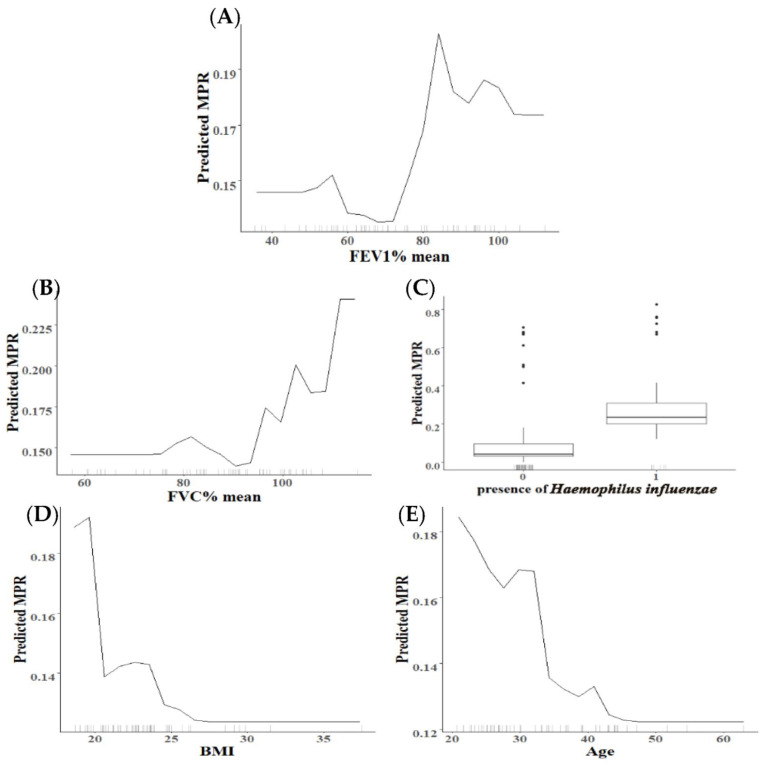
Partial dependence plots for the top 5 most important factors (panel (**A**): FEV_1_% mean, panel (**B**): FVC% mean, panel (**C**): presence of *Haemophilus influenzae*, panel (**D**): BMI, panel (**E**): Age) for low compliance.

Our results showed that most quantitative variable factors assessed in both models were not linearly associated (Figure 7 and Figure 8). For example, the positive non-linear effect of age on MPR > 80% compliance is observed when the age is below 50 years, but after that point, the effect is marginal (Figure 7C). In addition, there is an almost linear negative association between mean lung function (FEV_1_%) or the mean number of days with oral exacerbations and MPR > 80% (Figure 7A–D). Furthermore, qualitative variables assessed in the first model showed that the use of Ivacaftor/Tezacaftor treatment was positively related to compliance, as was the absence of methicillin-sensitive *Staphylococcus aureus* (MSSA).

For low compliance (MPR < 50%), there was a positive non-linear effect for lung function (Figure 8A,B) and a negative non-linear effect for BMI and age (Figure 8D,E). The categorical variable assessed in this model, the presence of *Haemophilus influenzae*, was also positively related to low compliance (Figure 8A,E).

## 3. Discussion

This is one of the first studies to longitudinally assess TA in CF, including CFTR modulator therapy among the different treatments evaluated. In addition, this study is also novel in terms of the application of new Machine Learning tools, specifically the creation of RF models, to assess which variables are most important in terms of compliance.

Overall, compliance was good for all CFTR modulators (Ivacaftor, Tezacaftor-Ivacaftor, and Elexacaftor-Tezacaftor-Ivacaftor), azithromycin, as well as aztreonam and tobramycin in inhalation solution. Compliance was moderate for the remaining treatments, and close to low compliance was observed with rhDNAsa. Among the inhaled antibiotics, colistin for inhalation and dry powder had the poorest MPR.

In our study, we observed a decrease in respiratory exacerbations, both oral and intravenous, during the study period, favored by isolation and prevention measures, hand washing and widespread use of masks during the pandemic [22,23].

We identified relevant predictors of high (MPR > 80%) and low compliance (MPR < 50%) from the generated RF model. Overall, we found that the top 5 variables contributing to high compliance were FEV_1_% mean, use of Ivacaftor/Tezacaftor, Age, mean number of days with oral exacerbations, and presence of methicillin-sensitive *Staphylococcus aureus*. On the other hand, for low compliance, the top predictors in the model were FEV_1_% mean, FVC% mean, presence of *Haemophilus influenzae*, BMI, and age.

A recent study on compliance over one year with the CFTR modulators Ivacaftor, Lumacaftor-Ivacaftor, and Tezacaftor-Ivacaftor analyzed a total of 2548 patients, 1289 (50.6%) patients with Lumacaftor/Ivacaftor, 784 (30.8%) with Ivacaftor and 475 (18.6%) with Tezacaftor/Ivacaftor. The mean MPR value for all CFTR modulators was higher than 80; they concluded the importance of monitoring TA due to the high cost of these treatments [24].

Classical articles assessing TA in CF demonstrate low compliance in CF patients [2,10,11,12,13,20,21,22,25]. Low TA is associated with a more marked decline in lung function and a higher number of exacerbations [2,10,11,12,13]. Adhesion is usually worse in adolescents and young adults [22], as we also saw in our results. However, in our work, TA was better than those described in the literature, in agreement with a recent publication by ManiKa K et al. [26], perhaps because we did not include the adolescent population. In this article, a 4-year longitudinal study of 55 patients enrolled in two centers, the MPR of inhaled antibiotic treatment was 0.75 ± 0.19, better than expected. Patients with a mean MPR ≥ 80% weighed statistically significantly more than those with moderate and low adherence, as in our study. *Haemophilus influenzae* chronic infections were associated with TA, and in CF, this pathogen is often associated with milder disease, perhaps not surprisingly associated with lower MPR [3].

Another interesting result of our study, in contrast to previous reports, was that TA was better (MPR ≥ 80%) in those patients with a higher number of exacerbations and a deterioration in FEV_1_% of 0.25. The group with moderate or low MPR had fewer exacerbations and an improvement in FEV_1_% of 7.1 and 1.6%, respectively, over the study period. The latter may be favored by the reduction of exacerbations observed in these two groups. These results contradict those previously described [10,11,12,13]; it can be postulated that this “more severe patient profile,” with more exacerbations and functional impairment is more informed of the consequences of the lack of TA. In recent times, there is a greater facility to access information, as well as a change in the doctor-patient relationship from a paternalistic model to a participative relationship, in which the patient is involved in decision-making and treatment, which may have resulted in an improvement in TA, compared to what was previously published [12,13,27,28,29].

Furthermore, the implementation of studies using these novel and statistical tools for the study of predictor factors allows for a deeper understanding of the knowledge of TA. Our results show that using machine learning algorithms, especially RF, is a promising methodology for analzying cross-sectional studies, showing robust predictive power and the ability to identify predictors of major importance. So far, this methodology has previously been used to evaluate factors with the greatest impact on high HIV viral load, COVID mortality, or presence of *Bovine Viral Diarrhoea Virus* [30,31,32], among others, but this is the first study of its application in CF and TA.

In most CF studies assessing TA, MPR has been used, comparing what is recorded in the hospital pharmacy with what should be consumed according to the prescription recorded by the physicians in the medical records [14,15]. This is a method that, although it has some limitations, is simple and comes close to the actual consumption of treatments. Within the multidisciplinary management of CF, as a multisystemic disease, the role of the hospital pharmacist is relevant. The pharmacist must coordinate and communicate closely with the medical team to provide the correct pharmacological prescription [33,34]. They can also provide us with TA information, a notable aspect to evaluate, among others, when deterioration or poor evolution is observed in a patient with CF. Likewise, although the declaration of the SARS-CoV-2 pandemic and the state of alert was a fortuitous event [22], as hospital pharmacies arranged mechanisms to deliver medication to patients’ homes, no deterioration in TA was observed [33,34,35]. On the other hand, the pharmaceutical cost of the treatments required by CF patients is usually very high, and adequate adherence is necessary to obtain the expected results [36]. With the advent of CFTR modulators, where the cost is even higher, adequate adherence is required to optimize their benefit fully. This is why the pharmacist’s participation in the multidisciplinary team for managing and following up with CF patients is a priority [36]. The role of pharmacists in CF units is a priority because they can give us information on MPR that allows us to improve TA. The lack of adherence in chronic treatments (oncohematological, viral pathologies, multiple sclerosis, chronic inflammatory diseases, mental health, transplanted patients, minority respiratory diseases) is a universal and real problem that has very important clinical, economic and social consequences justifying the work to address such a problem.

It is crucial that patients and caregivers know about the disease and its consequences for a better TA. Patients who understand the relevance of adherence to therapy and who know the importance of its treatments are more likely to be adherent. Health education and support to patients and families by a multidisciplinary team (doctors, pharmacists, nurses, psychologists...) must be a priority objective to convey the importance of treatments in an easy and comprehensive way. The use of different technological tools can help motivate patients to follow treatments. One strategy is to establish daily and weekly routines agreed with the patient to ensure that treatments are carried out and to provide organizational strategies appropriate to the age and psychosocial environment. Data obtained are not consistent with the literature and may be supported by education and constant assistance to transmit the importance of compliance.

It is true that with the advent of ETI [7,8], patients’ lives have improved substantially, reducing cough, expectoration, and exacerbations and, improving digestive symptomatology and nutritional status, increasing lung function and quality of life. Thus, the onset of ITD provides an opportunity to consider reductions in the overall treatment burden [37] and assess whether other chronic medications can be safely discontinued without loss of clinical benefit. Studies have been designed to provide the answer, the Simplify study [38] and CF-Storm [39]. Simplify is a US protocol to evaluate the impact of discontinuation versus continuation of hypertonic saline or rhDNase in people with CF who are at least 12 years or older and stable on ETI therapy. The primary objective of each trial is to determine whether discontinuation of therapy is non-inferior to the continuation of therapy after the establishment of ETI, as measured by the 6-week absolute change in FEV_1_% [38] (NCT04378153). A similar study is being conducted in the UK CF STORM [39]. The results will help us make decisions on simplifying and optimizing mucociliary clearance therapies without risk to our patients.

Our work has some limitations. We have not evaluated the totality of medication, such as pancreatic enzymes or antibiotics required during exacerbations, or compliance with physiotherapy techniques or physical exercise. Furthermore, we only included adult patients; perhaps if there had been an adolescent population, the TA would have been lower. MPR provides objective information relevant to assessing TA. We start from the assumption that if patients pick up medication from the pharmacy, they will take it as prescribed. On the other hand, during the study, a state of alarm was declared, and the fact that the pharmacy took the medication home and that, due to social isolation and teleworking measures, more time was available to carry out the treatment could have led to a bias. However, we consider it an important study because it encompasses a non-negligible number of well-characterized patients, assesses TA longitudinally, including both symptomatic treatments and CFTR modulators, and implements novel analyses on predictors of TA. Measuring adherence helps us know the patients we need to work with to reinforce the importance of adherence and improve it for better results.

In conclusion, TA in adult CF patients was good to moderate, better than previously described. All CFTR modulators, azithromycin, aztreonam, and tobramycin in inhalation solution, had good TA. CF treatments are expensive, so we need to evaluate TA, and analysis of predictors of TA helps to investigate the issue further and obtain maximum clinical benefit. Factors such as age, lung function, respiratory exacerbations, chronic bronchial infection by some pathogens, and BMI are associated with TA. The role of the pharmacist in CF units is a priority; among other things, they can give us information on MPR that allows us to improve TA. Knowing the degree of adherence of each patient at all times and for each medication helps us to individualize appropriate strategies such as simplifying treatment, information, and health education. All this will have a positive impact on the control of their disease. We are experiencing a change in the lives of CF patients with the introduction of CFTR modulatory therapies, which may be an opportunity to optimize and simplify our patients’ treatments. However, we must wait for the publication of studies that guarantee their safe use.

## 4. Materials and Methods

### 4.1. Patient Recruitment

Patients over 18 years of age with a CF diagnosis, who were being monitored in the Adult Unit of the Hospital Universitario de la Princesa and were given medication at the Pharmacy of the Hospital de la Princesa. Patients being monitored from 1 January 2019 to 30 June 2021 were included. The adult CF unit works in coordination with the pediatric unit of the Niño Jesús Hospital, with patients being transferred at 18 years of age and being assessed in the transitional consultations located in the pediatric unit by the adult pulmonologist and the pediatrician between 16 and 18 years of age. We excluded patients who picked up medication at other pharmacies outside the Community of Madrid and those who had transferred from the pediatric or the other units in the last six months because they could have unquantified medication at home, which could lead to miscalculations. Patients were included consecutively during the third quarter of the year 2021 as patients were seen in the CF unit consultation and met the inclusion criteria for the study. Inclusion criteria: Patients diagnosed with CF (entitled to the pharmaceutical provision by the Madrid Health Service) monitored at the Princess Unit and who had signed the informed consent of the European Registry. Exclusion criteria: Those who belonged to or moved to other autonomous communities in the study period, patients recently transferred to the Hospital de la Princesa Unit, and those referred for lung transplantation. All clinical and MPR data were reviewed retrospectively over the time period studied. Patients had signed a generic informed consent form (CF patient registry) in which they authorized us to use their data anonymously and in accordance with the regulations. The project was approved by the ethics committee Registration Nr 4975 (document attached).

An ambispective observational study was carried out on the cohort of patients, and the patients’ AT to the different pharmacological treatments prescribed was analyzed, substances to improve mucociliary clearance (rhDNase and hypertonic saline), aerosol antibiotics (specific and intravenous formulations), azithromycin, vitamins, and CFTR modulators, Ivacaftor, was introduced in Spain in December 2015 and Lumacaftor-Ivacaftor, Ivacaftor-Tezacaftor and Elexacaftor-Texacaftor-Ivacaftor as compassionate use in critically ill patients from December 2019.

Patients were contacted by phone or email requesting the medication they needed each month and came to the hospital to pick it up. In March 2020, the SARS-CoV-2 pandemic and state of alert were declared, and different mechanisms were arranged to avoid exposure and risk of contagion to the virus [22,23]. Different methods were arranged to send the requested medication to the patient’s homes to avoid unnecessary visits and to ensure that patients continued to take their medication. The distribution system for treatments to patients’ homes has been maintained to the present day.

### 4.2. AT Assessment and Collection of Clinical Variables

The assessment of TA was calculated using the MPR index, which was defined as the sum of all days of medication supply received, divided by the number of days the medication was prescribed during the study period using the equation [(days covered with dispensed medication/replacement interval) × 100] [14]. Data were obtained from the electronic records of the hospital pharmacy, which were cross-checked against the electronic history of prescriptions prescribed by the responsible physician. Adherence was considered good if it was greater than or equal to 80%, moderate between 50–<80%, and low <50% [14,15].

The following clinical variables were collected: age and BMI at baseline, sex, CFTR gene mutation, exocrine pancreatic insufficiency, CF-related diabetes (receiving insulin or oral antidiabetic treatment), chronic bronchial infection (defined by the presence of the same microorganism in 50% of sputum samples collected in 1 year), history of allergic bronchopulmonary aspergillosis and massive hemoptysis (more than 240 mL in 24 h or 100 mL in 1 h), the best FEV_1_% in each year of the study and the number of respiratory exacerbations per year, an exacerbation is considered the need to prescribe additional antibiotic treatment in the presence of clinical changes, with the number of courses of oral antibiotics in the case of mild or moderate exacerbations or intravenous antibiotics in the case of severe exacerbations being collected.

Low, moderate, or high MPR was related to age, sex, chronic bronchial infection, lung function, 2.5-year FEV_1_% decline rate, and a number of exacerbations, whether treated with oral or intravenous antibiotics.

### 4.3. Statistical Analysis

First, a descriptive analysis of the characteristics of the total patients and their total compliance during the study was performed, calculating the mean and standard deviation of the quantitative variables. The Shapiro-W Kolmogorov-Smirnov test was performed to check the normality of continuous variables. Homoscedasticity was tested using Levene’s test. When the distributions were normal and homoscedastic, a parametric test (Anova, *t*-test) was performed. When one of these two assumptions was not met, a non-parametric test (Kruskal-Wallis test, Mann-Whitney-Wilcoxon test) was performed. In the case of qualitative variables, the comparison of proportions was tested using the chi-square test or Fisher’s exact test whenever necessary.

In addition, an RF analysis was conducted to assess the relative importance of explanatory variables in the classification of the compliant (MPR > 80%) and non-compliant (MPR < 50) patient profiles. The importance of the variables was assessed through the Mean Decline in Accuracy (MDA) and Mean Decline in Gini (MDG) indicators, although to assess the importance of the variables, we focused mainly on the use of the MDG parameter since the classifications produced by this index provide more robust results [25]. The higher value of MDG implies that they are the most important predictors in the model. All analyses will be carried out using R statistical software.

## Figures and Tables

**Table 1:** Demographic and clinical characteristics for the total number of patients and for each compliance group.

Demographic and Clinical Characteristics	Total (*n* = 57)	MPR < 50 (*n* = 8)	MPR 50–80 (*n* = 19)	MPR > 80 (*n* = 30)	*p*-Value
Age, (mean ± SD)	33.9 ± 9.4	28.5 ± 6.3	32.0 ± 10.5	36.5 ± 8.7	0.030 *^4^
20–30 yrs., *n* (%)	25 (43.9%)	6 (75.0%)	11 (57.9%)	8 (26.7%)	0.017 *
30–40 yrs., *n* (%)	16 (28.1%)	1 (12.5%)	4 (21.1%)	11 (36.7%)	0.421
≥40 yrs., *n* (%)	16 (28.1%)	1 (12.5%)	4 (21.1%)	11 (36.7%)	0.421
Gender, *n* (%)					
Women	34 (59.6%)	4 (50.0%)	11 (57.9%)	19 (63.3%)	0.810
BMI, (mean ± SD)	23.2 ± 3.4	22.1 ± 1.7	23.7 ± 4.5	23.2 ± 2.9	0.786
Genotype, *n* (%)					
F508del/F508del	20 (35.1%)	1 (12.5%)	5 (26.3%)	14 (46.7%)	0.145
F508del/other	23 (40.4%)	4 (50.0%)	11 (57.9%)	8 (26.7%)	0.072
other/other	14 (24.6%)	3 (37.5%)	3 (15.8%)	8 (26.7%)	0.429
Pancreatic insufficient, *n* (%)	46 (80.7%)	6 (75.0%)	15 (78.9%)	25 (83.3%)	0.727
Diabetes, *n* (%)	24 (42.1%)	4 (50.0%)	8 (42.1%)	12 (40.0%)	0.933
Allergic bronchopulmonary aspergillosis, *n* (%)	14 (24.6%)	2 (25.0%)	3 (15.8%)	9 (30.0%)	0.529
Massive hemoptysis, *n* (%)	9 (15.8%)	1 (12.5%)	2 (10.5%)	6 (20.0%)	0.783
Lung function, (mean ± SD)					
FEV_1_^1^ %					
2019	71.0 ± 20.3	80.5 (16.8)	75.4 (23.7)	65.7 (17.8)	0.099
2020	72.9 ± 18.5	84.1 (15.7)	76.4 (22.2)	67.6 (15.1)	0.042
2021 ^3^	71.8 ± 18.7	82.0 (10.2)	76.4 (22.5)	66.9 (16.1)	0.059
Total	72.2 ± 18.9	81.7 (15.1)	76.4 (22.5)	66.9 (16.1)	0.069
FVC% ^2^					
2019	82.7 ± 15.6	90.0 (13.0)	86.1 (14.7)	78.5 (15.9)	0.094
2020	88.1 ± 14.0	99.3 (11.6)	90.6 (14.1)	83.5 (12.7)	0.009 *
2021 ^1^	87.9 ± 13.6	96.3 (12.9)	91.1 (13.8)	84.2 (12.7)	0.059
Total	86.5 ± 13.9	95.0 (11.6)	89.6 (13.8)	82.2 (13.3)	0.031
Bacteria presence, *n* (%)					
Chronic bronchial infection	48 (84.2%)	8 (100%)	16 (84.2%)	24 (80.0%)	0.593
*Pseudomonas aeruginosa*	22 (38.6%)	1 (12.5%)	6 (31.6%)	15 (50.0%)	0.377
methicillin-sensitive *Staphylococcus aureus* (MSSA)	16 (28.1%)	4 (50.0%)	7 (36.8%)	5 (16.7%)	0.511
methicillin-resistant *Staphylococcus aureus* (MRSA)	10 (17.5%)	2 (25.0%)	3 (15.8%)	5 (16.7%)	0.951
*Achromobacter xylosoxidans*	6 (10.5%)	1 (12.5%)	3 (15.8%)	2 (6.7%)	0.921
*Burkholderia cepacia*	5 (8.8%)	1 (12.5%)	1 (5.26%)	3 (10.0%)	0.915
*Aspergillus sp*	1 (1.8%)	0 (0.0%)	0 (0.0%)	1 (3.3%)	0.806
*Haemophilus influenzae*	6 (10.5%)	4 (50.0%)	0 (0.0%)	2 (6.7%)	0.004 *
*Stenotrophomonas maltophilia*	0 (0.0%)	0 (0.0%)	0 (0.0%)	0 (0.0%)	1.000
Mycobacteria non tuberculous	1 (1.8%)	0 (0.0%)	0 (0.0%)	1 (3.33%)	0.806

^1^ Forced Expiratory Volume in 1 s. ^2^ Forced Vital Capacity. ^3^ Time considered between January to July 2021. ^4^ Statistical significant differences are marked with an asterisk (*).

**Table 2:** Mean number of respiratory exacerbations with oral and intravenous cycles in each year of the study and in total.

Year		Oral Administration					Intravenous Administration				
		Total (*n* = 57)	MPR < 50 (*n* = 8)	MPR 50–80 (*n* = 19)	MPR > 80 (*n* = 30)	*p*-Value	Total (*n* = 57)	MPR < 50 (*n* = 8)	MPR 50–80 (*n* = 19)	MPR > 80 (*n* = 30)	*p*-Value
**Exacerbations** **(mean ± SD)**	**2019**	2.1 ± 1.9	1.6 ± 1.2	2.2 ± 1.7	2.1 ± 2.1	0.763	0.4 ± 0.8	0.0 ± 0.0	0.4 ± 0.9	0.5 ± 0.9	0.220
	**2020**	1.8 ± 1.6	1.5 ± 0.9	1.4 ± 1.6	2.1 ± 1.7	0.228	0.2 ± 0.4	0.0 ± 0.0	0.3 ± 0.5	0.3 ± 0.5	0.217
	**2021**	0.9 ± 1.0	0.9 ± 1.2	0.9 ± 0.9	0.8 ± 1.0	0.858	0.0 ± 0.1	0.0 ± 0.0	0.0 ± 0.0	0.0 ± 0.2	0.648
	**Total**	1.5 ± 1.1	1.4 ± 0.7	1.5 ± 1.1	1.7 ± 1.3	0.71	0.2 ± 0.3	0.0 ± 0.0	0.2 ± 0.3	0.3 0.4	0.072
**≥1 exacerbation, n (%)**	**2019**	43 (75.4%)	6 (75.0%)	15 (78.9%)	22 (73.3%)	0.905	14 (24.6%)	0 (0.0%)	5 (26.3%)	9 (30.0%)	0.211
	**2020**	43 (75.4%)	7 (87.5%)	12 (63.2%)	24 (80.0%)	0.285	14 (24.6%)	0 (0.0%)	5 (26.3%)	9 (30.0%)	0.211
	**2021**	31 (54.4%)	3 (37.5%)	12 (63.2%)	16 (53.3%)	0.467	1 (1.8%)	0 (0.0%)	0 (0.0%)	1 (3.3%)	0.633
	**Total**	42 (73.7%)	7 (87.5%)	14 (73.7%)	21 (70.0%)	0.607	4 (7.0%)	0 (0.0%)	1 (5.3%)	3 (10.0%)	0.576
**Days ^2^** **(mean ± SD)**	**2019**	47.9 ± 32.5	33.7 (10.1)	48.5 (24.4)	51.1 (39.9)	0.548	**26.6 ± 14.0** ^1^	NA ^3^ ± NA	26.6 ± 17.4	**26.6** **±** **12.9**	0.838
	**2020**	42.2 ± 31.0	30.0 (18.4)	37.9 (23.8)	47.9 (36.2)	0.452	**16.2 ± 4.7**	NA ± NA	18.2 ± 3.8	**15.1** **±** **5.0**	0.246
	**2021**	29.5 ± 18.6	35.0 (18.5)	21.6 (11.3)	34.3 (21.8)	0.137	14.0 ± ND	NA ± NA	NA ± NA	14.0 ± NA	NA
	**Total**	30.8 ± 22.4	22.9 (12.4)	28.9 (16.1)	34.2 (27.1)	0.568	9.7 ± 5.5	NA ± NA	10.7 ± 4.2	9.3 ± 6.2	0.275

^1^ The statistically significant difference (*p* < 0.05) found for a determined variable over time (2019–2020) was marked in bold. ^2^ Considering the days from those patients that had at least one exacerbation. ^3^ Data not available.

**Table 3:** Associated Mean Decreases Accuracy (MDA) and Mean Decrease Gini (MDG) of high-importance predictors of MPR > 80 and MPR < 50.

Variable	MPR > 80		MPR < 50	
	MDA	MDG	MDA	MDG
**Gender**	−1.1	0.5	0.3	0.3
**Age**	0.5	3.0	−0.1	1.3
**Genotype**	2.8	1.9	−2.1	0.4
**Pancreatic insufficient**	0.0	0.1	−0.1	0.1
**Diabetes**	1.6	0.5	−1.6	0.2
**Allergic bronchopulmonary aspergillosis**	0.1	0.5	0.4	0.2
**Massive haemoptysis**	−1.1	0.6	−2.1	0.1
**BMI**	0.3	1.8	−0.9	1.4
**Chronic bronchial infection**	0.1	0.3	1.9	0.1
***Pseudomonas aeruginosa***	0.0	0.1	−0.1	0.1
**methicillin-sensitive *Staphylococcus aureus* (MSSA)**	1.1	2.5	0.2	0.3
**methicillin-resistant *Staphylococcus aureus* (MRSA)**	0.0	0.0	0.0	0.3
***Achromobacter xylosoxidans***	0.0	0.3	−0.2	0.2
***Burkholderia cepacia***	0.0	0.0	−0.1	0.1
***Aspergillus sp***	0.0	0.0	0.0	0.0
***Haemophilus influenzae***	0.0	0.0	5.7	1.4
**Mycobacteria non tuberculous**	0.0	0.3	0.0	0.1
**Total exacerbations by intravenous administration**	0.0	0.1	0.0	0.0
**Total exacerbations by oral administration**	−1.1	0.7	0.1	0.1
**Hypertonic saline solution**	1.1	0.2	3.7	1.0
**DNAsa**	−0.9	0.3	3.3	0.5
**Azithromycin**	0.0	0.4	1.7	0.3
**Aztreonam solution for inhalation**	1.1	0.4	0.1	0.1
**Tobramycin in solution for inhalation**	1.5	0.3	−0.8	0.0
**Colistin dry powder**	−1.6	0.6	0.6	0.1
**Colistin in solution for inhalation by INEB**	0.0	0.0	0.8	0.1
**Ivacaftor**	0.0	0.0	0.0	0.1
**Ivacaftor/Tezacaftor**	−0.2	3.0	1.7	0.1
**Elexacaftor, Tezacaftor/Ivacaftor**	0.0	0.0	1.0	0.0
**Vitamins**	0.0	0.0	0.9	0.2
**Intravenous formulation**	0.0	0.1	−0.8	0.1
**Total FVC%**	1.6	2.1	2.3	1.4
**Total** **FEV_1_** **%**	1.6	3.3	2.0	1.4
**Total days of intravenous exacerbations**	0.8	1.0	0.2	0.3
**Total days of oral exacerbations**	0.9	3.0	0.6	0.9

## Data Availability

The data presented in this study are available on request from the corresponding author. The data are not publicly available.
